# Feasibility of Collecting Diary Data From Asthma Patients Through Mobile Phones and SMS (Short Message Service): Response Rate Analysis and Focus Group Evaluation From a Pilot Study

**DOI:** 10.2196/jmir.6.4.e42

**Published:** 2004-12-02

**Authors:** Jacob Anhøj, Claus Møldrup

**Affiliations:** ^2^The Danish University of Pharmaceutical SciencesDepartment of Social PharmacyCopenhagenDenmark; ^1^H:S Unit for Patient SafetyHvidovreDenmark

**Keywords:** Internet, cellular phone, asthma, disease management, patient compliance, focus groups, qualitative research

## Abstract

**Background:**

Self-management of asthma may improve asthma outcomes. The Internet has been suggested as a tool for the monitoring and self-management of asthma. However, in a recent study we found that a Web interface had some disadvantages and that users stopped using the application after a short while.

**Objective:**

The primary objective of this study was to evaluate, from a user perspective, the feasibility of using short message service (SMS) for asthma diary data collection through mobile phones. The secondary objective was to investigate patient compliance with an SMS diary, as measured by response rates over time.

**Methods:**

The study included quantitative response rate data, based on SMS collection, and qualitative data from a traditional focus group setting. In a period of 2 months, the participants received 4 SMS messages each day, including a medication reminder, a request to enter peak flow, data on sleep loss, and medication dosage. Participants were asked to reply to a minimum of 3 of the messages per day. Diary inputs were collected in a database and the response rate per patient was expressed as the number of diary inputs (SMS replies) divided by diary requests (product of number of days in the study and the number of diary questions per day) for each participant. After the study period, the participants were invited to a focus group interview addressing the participants' attitudes to their disease, their experience with the SMS asthma diary, and their future expectations from the SMS asthma diary.

**Results:**

Twelve patients with asthma (6 males, 6 females) participated in the data collection study. The median age was 38.5 (range: 13 – 57) years. The median response rate per patient was 0.69 (range: 0.03 – 0.98), ie, half the participants reported more than about two thirds of the requested diary data. Furthermore, response rates were relatively steady during the study period with no signs of decreasing usage over time. From the subsequent focus group interview with 9 users we learned that, in general, the participants were enthusiastic about the SMS diary – it became an integrated part of their everyday life. However, the participants wished for a simpler diary with only one SMS message to respond to and a system with a Web interface for system customization and graphical display of diary data history.

**Conclusion:**

This study suggests that SMS collection of asthma diary data is feasible, and that SMS may be a tool for supporting the self-management of asthma (and possibly other chronic diseases) in motivated and self-efficacious patients because mobile phones are a part of people's everyday lives and enable active requests for data wherever the patient is. The combination of SMS data collection and a traditional Web page for data display and system customization may be a better and more usable tool for patients than the use of Web-based asthma diaries which suffer from high attrition rates.

## Introduction

The cornerstone of modern asthma care is self-management, allowing the patients to monitor their disease severity continuously and to adjust the dose of inhaled corticosteroid based on symptoms, lung function and the use of rescue medication [1]. A recent Cochrane Review concluded that self-management might improve asthma outcomes significantly [2]. Several strategies have been developed to support self-management, including patient education and written action plans. In a recent qualitative study on patients' and doctors' experience with a Web-based asthma diary, LinkMedica Asthma [3], we found that patients and doctors were first enthusiastic about the diary in general. This study allowed doctors to access patient diary data online, thus facilitating the cooperation between doctors and patients. However, we also identified severe problems of diary maintenance over time, mainly due to lack of integration of Internet use into users' everyday life [4]. We suggested the exploration of other technologies for diary data collection. The main requirement of such technology would be that the system actively requests data from the user instead of passively waiting for the user to enter data. The diary/user interaction should be initiated by the system – not by the user, thus helping users to remember to fill in their diary and making frequent access to the diary as easy as possible. The use of reminders (emails, phone calls, mobile phone text messages or letters) to improve patient behaviour is well documented within several areas, eg, vaccination [5], the use of oral contraceptives [6], the use of prescription medicines [7], and attendance rates [8].

A number of data collection and reporting technologies would be of interest in order to seamlessly integrate this process into the users' everyday lives and to initiate the interaction with the user, eg, Short Message Service (SMS), General Packet Radio Service (GPRS), Wireless Application Protocol (WAP), and Personal Digital Assistants (PDAs). We decided to explore the oldest and most commonly available of these, SMS, as we believed that a key factor for success would be availability and the users' familiarity with the technology. It was assumed that more than 80% of Danes own a mobile phone [9]. Another advantage of SMS was that the users already had the technology (mobile phones) and that no development of client software would be necessary.

SMS is a service for sending messages of up to 160 characters (224 characters, if using a 5-bit mode) to mobile phones that use Global System for Mobile (GSM) communication. GSM and SMS services are primarily available in Europe. SMS is similar to paging. However, SMS messages do not require the mobile phone to be active and within range and will be held for a number of days until the phone is active and within range.

This study explored the use of the mobile telephone and SMS as an appropriate interface for asthma diary data collection.

The primary objective of this study was to evaluate the feasibility of SMS for asthma diary data collection from a patient/user perspective. The secondary objective was to investigate response rates over a period of 2 months from SMS collection of asthma diary data in asthma patients. It was not an objective to investigate the effect of SMS diary data collection on clinical asthma outcomes, which is an important question, but beyond the scope of this pilot study.

## Methods

This study included the collection of quantitative usage (response) data, based on SMS asthma diary data collection, and qualitative data from a traditional focus group setting [10].

### Participant Recruitment

A convenience sample of self-selected participants was recruited from the Danish website LinkMedica Asthma [3] between 24 February and 25 March 2003. When visiting LinkMedica Asthma during this period, users were presented with a pop-up window asking if they were interested in participating in a test of asthma diary data collection using SMS. Except for the introductory text, a “yes” and a “no” button were the only elements on the pop-up window. If the user clicked the “no” button, the window closed. If the user clicked the “yes” button, an email address was displayed and the user was encouraged to contact this email address for more information. Regardless of the button clicked, a cookie was set on the user's hard disk to prevent the pop-up window from appearing at revisits. If the pop-up window was closed by other means (eg, by clicking the cross in the top right corner), no cookie was set.

The response rate of the pop-up was not monitored. But the number of respondents who indicated interest in the study was compared to the number of hits on the pop-up page in the same period (see Results).

Respondents then received a letter with information about the study and a short questionnaire about their background and their prior use of LinkMedica Asthma; those who responded to this were included in the study. Since the focus of the study was the SMS interface rather than the disease itself, we did not attempt to verify the diagnosis of asthma, nor did we interfere with the participants' current treatment.

The participants used their own mobile phones during the study and they did not receive any reimbursement of expenses for SMS messages, which were in the order of DKK 50 (Danish Kroner; about US $8.78, or 7.72 EUR). However, after completion of the study the participants received a gift voucher of DKK 500. They were not informed about this in advance.

### Design of SMS Diary

During the 2-month study period (from 12 December 2003 to 15 February 2004) the participants received a sequence of text messages each day at a self-selected time of the day:

1. Remember to take your controller medication.
2. Remember to measure your peak flow – what was your peak flow?
3. Were you awake during the night due to asthma symptoms?
4. How many doses of your rescue medication have you taken during the last 24 hours?

**Figure 1:**
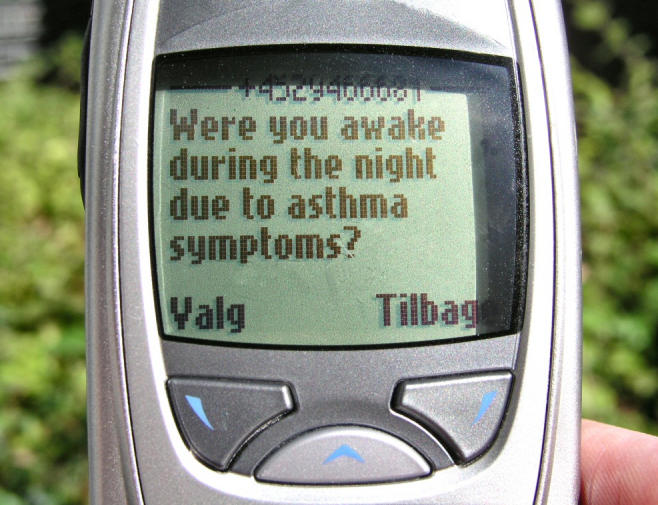
An example of an SMS-message; the user is supposed to reply with an appropriate answer, in this case “yes” or “no”.

An example of an SMS-message is displayed in Figure 1.

Three of these questions (questions 2, 3, and 4) required a response and are referred to as “diary questions”. The participants were expected to answer these diary questions by replying with an appropriate answer: peak flow (L/min.), yes or no, and number of doses respectively. Messages number 3 and 4 were sent only if the user had answered questions 2 and 3 respectively. The delay between the users' reply to one message and the reception of the next message was approximately 1 minute. This was the time needed for the reply message to reach the server, the server to validate the reply and send out the next message and for the next message to reach the user.

The diary questions were the same as previously used in the Web-based LinkMedica Asthma diary. They were devised by LinkMedica's advisory board. As recently reported, usage of this Web-based asthma diary was found to be suboptimal as users were not using the site for more than short periods of time. The primary reason for this appeared to be that LinkMedica did not fit into their everyday lives because of technical and psychological aspects [4].

A database system collected information about diary requests and inputs (ie, user ID, question ID, date and time of request, date and time of input, and input value).

Two participants (user IDs 2 and 7) who had not used peak flow measuring prior to the study did not receive the second message about peak flow, because we did not want the study to interfere with their current routines.

In contrast to the asthma diary on LinkMedica Asthma, the SMS diary did not provide any feedback to participants on asthma status on the basis of their diary values. Thus, it was stressed to the participants before entering the study that they themselves were responsible for consulting their general practitioner or their asthma clinic if their asthma deteriorated.

### Analysis of Usage Data

Usage data was analysed with R Statistical Software version 1.8.1 [11] and are reported as response rate per patient and per day:

- Response rate per patient: Total number of diary inputs (SMS replies) divided by diary requests (product of number of days in study and number of diary questions per day) for each participant
- Response rate per day: Number of diary inputs divided by number of requests for each study day.

We also looked at the total number of days where users replied to all, some or none of the diary questions. A study day with full usage is a day where the user answered all (two or three) questions in the diary. A study day with partial usage is a day where the user answered some (one or two), but not all questions in the diary.

### Focus Group Interview

The focus group interview was done on 23 February 2004 and videotaped for subsequent qualitative analysis. Three themes were addressed during the interview:

- How participants related to their asthma in general
- The participants' experience with the SMS asthma diary
- The participants' future expectations from the SMS asthma diary.

## Results

### Participant Recruitment

During the recruitment period, the recruitment pop-up was shown 1317 times. Fifteen persons responded with “yes” (indicating interest in study participation) and were sent, via the mail, an information letter with a questionnaire. Twelve (6 males and 6 females) responded and were included in the study. The median age was 38.5 (13 to 57) years. Geographically, the participants represented both urban and rural areas of Denmark and one came from Sweden. Nine of these individuals participated in the subsequent focus group interview, which was held 1 week after the study period ended.

The self-reported prior experience with SMS messages (as determined in the subsequent focus group) was moderate for the majority of the participants for whom data were available. One participant had never used SMS prior to this study. Five participants were medium users receiving and sending 1 to 3 messages daily. Three participants were heavy users receiving and sending more than 4 messages daily. Prior to this study, none of the participants had used a questionnaire based on SMS. However, in general the mobile phone was an integrated part of the everyday life of all participants.

### Diary Usage

**Figure 2:**
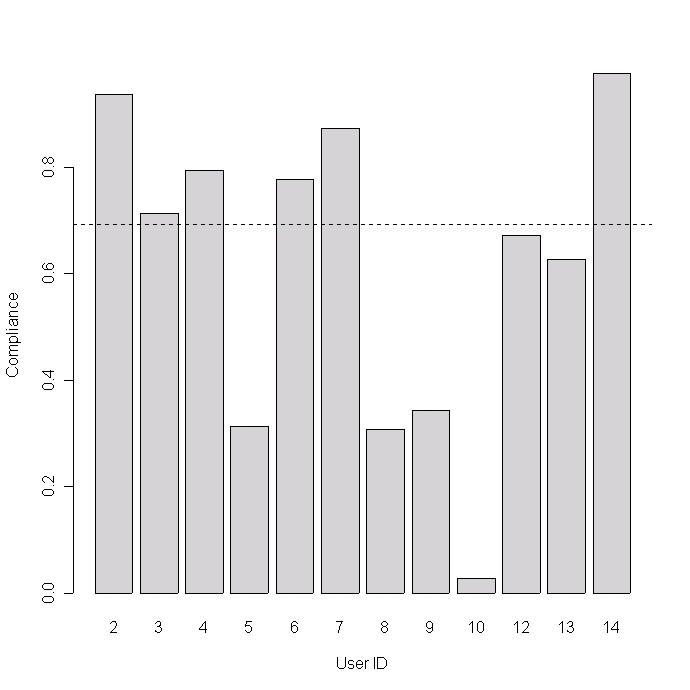
Diary response rate per patient: total number of diary inputs (SMS replies) divided by diary requests (product of number of days in study and number of diary questions per day) for each participant. Numbers are shown in Table 1. The horizontal line marks the median compliance (0.69).

The median response rate per patient was 0.69, ranging from 0.03 to 0.98, ie, half the participants replied to more than two thirds of the requested diary data. Four participants had a low response rate – less than 0.4 (and did not attend the focus group meeting) – while the rest had response rates greater than 0.6 (Table 1, Figure 2).

**Figure 3:**
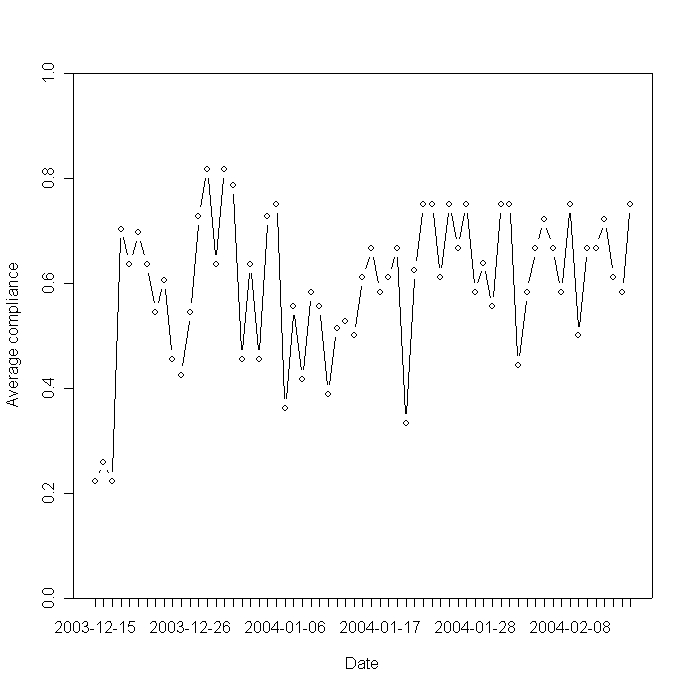
Response rate per day in the study period: number of diary inputs divided by number of requests for each study day

Apart from relatively low response rate in the days around Christmas and New Year's Eve, response rate per day, ie, number of diary inputs divided by number of diary requests, was relatively steady during the study period with no apparent signs of declining usage over time (Figure 3).

Out of a total of 727 study days, there were 423 days (58%) where users replied to all diary questions, 31 days (4%) where users replied to some diary questions, and 273 days (38%) where users did not reply at all.

### Focus Group Interview

#### How Participants Related to Their Asthma in General

In the focus groups, asthma was described as a burden requiring constant awareness and daily routines in order to gain and maintain control of the disease. Although the participants were aware that they had a chronic disease, they regarded their disease as something that occurs periodically like the flu or similar conditions.

In general, the participants agreed that their goal was to be able to handle everyday life without any symptoms. In contrast to what is recommended in current asthma care guidelines that emphasize the importance of self-care and awareness [1], they strived to create a stable situation taking the asthma disease out of focus thereby making them feel free from restrictions.

The participants also reflected on the patient/doctor relationship. It was evident that the participants found this relationship to be of extreme importance, but they also found that most doctors had limited interest in their disease. As one participant expressed:

Patient 14:* “…he [the general practitioner] may have all the information but lacks the continuous insight in my situation…”*

Some of the participants had previously participated in clinical research. They explained that the purpose of this was to gain more knowledge about asthma and, hopefully, to improve their own treatment.

**Table 1:** User demographics and response rate for the diary questions (user ID 1 and 11 were used for testing purposes and are not shown)

**User ID**	**Sex**	**Age (Years)**	**SMS Use Prior to the Study (per Day)**	**Days in Study**	**Number of Diary Questions per Day**	**Total Number of Diary Requests in Study Period**	Response Rate*
2	Male	48	0	63	2†	126	0.94
3	Female	40	1-3	63	3	189	0.71
4	Male	37	4-10	63	3	189	0.79
5	Female	57	1-3	63	3	189	0.31
6	Female	42	1-3	63	3	189	0.78
7	Female	22	> 10	63	2†	126	0.87
8	Female	19	NA‡	63	3	189	0.31
9	Female	34	NA‡	63	3	189	0.34
10	Male	30	NA‡	63	3	189	0.03
12	Male	13	4-10	59	3	177	0.67
13	Male	50	1-3	59	3	177	0.63
14	Male	51	1-3	42	3	126	0.98

^*^ Response rate per patient was calculated as the total number of diary inputs (SMS replies) divided by diary requests (product of number of days in study and number of diary questions per day) for each participant.

^†^ Two participants (user IDs 2 and 7) had not used peak flow measuring prior to the study and thus did not receive question #2 about peak flow, because we did not want the study to interfere with the participants' current routines.

^‡^ NA: Data not available (user not present at focus group interview).

#### Participants' Experience With the SMS Asthma Diary

On the negative side, the participants reported technical issues: Why three messages every day, instead of just one? Why was there a waiting time of 1 minute between each text message? Why did the system not provide feedback when diary data indicated lack of asthma control? The messages were delivered at the same time of the day during weekends and holidays, and some participants preferred not to be awakened early on their days off. Thus, all negative issues were attributable to the fact that the SMS diary system was in the early stages of development.

The positive aspects, which were predominant, were related to the feeling of control and support provided by the SMS diary. Suddenly the participants did not have to use their energy to remember to take their medication or enter diary data – the SMS system took care of this. All participants felt that the system positively influenced their ability to cope with the disease. One expressed this as follows:

Patient 4: “*…I am not good at routines. Therefore, it is great to get a reminder saying, ´take your medication.' It gives me freedom and creates control.*”

Some participants mentioned that for some purposes they preferred the daily support of the SMS diary to frequent personal support from a health care professional. It was not that they did not want to see their doctor or their asthma nurse for regular check-ups; but frequent (daily) self-monitoring using a computer system is less demanding and less insistent than frequent contact with a professional.

After the test period, several participants expressed the feeling of missing something. They missed the reminders and the diary questions. Some participants called the technical provider to report the missing text messages as a system breakdown. It was a general experience that the SMS diary became an integral part of the participants' everyday lives.

#### Participants' Future Expectations From the SMS Asthma Diary

The participants asked for a more flexible, dynamic, and customizable system. It is important that the patient – not the system – dictates how the SMS interface should behave. Some participants liked the daily “remember your medication” notices, others found them annoying. Some did not use peak flow measuring as part of their asthma monitoring every day and would have preferred to be able to switch peak flow measures on and off as needed. Some would have liked the system to work periodically, eg, in case of asthma exacerbation or before scheduled doctor visits instead of continuously. The participants agreed that the system should be much more interactive and responsive. If reported data indicate poor asthma control, the system should respond with an alert message or with a recommendation to see the doctor. The participants also suggested that the system be integrated with an Internet interface so there would be an opportunity to review the asthma symptoms over a period of time and options to customize the system to one's personal preferences. At best, the system should be integrated with the doctor's office either through the Internet or just by emails. Some participants suggested the use of picture messaging as part of the service.

## Discussion

In this study, we found that for a highly motivated and (self-)selected, but geographically and age diverse group of asthma patients, SMS collection of asthma diary data is feasible. In general, the participants were enthusiastic about the SMS diary. They suggested development of a live system combining an interactive SMS diary that included feedback messages with a personal Web page. The Web page should allow customization of the service to the preferences of the individual user and provide graphing and aggregation facilities for presentation of long-term diary data.

### Limitations of External Validity

The participants represented a diverse group of asthma patients in terms of age, sex, and prior usage of SMS. But the participants were also a homogeneous group in the sense that they were all concerned with their asthma disease status, otherwise they would not have volunteered for the study. The sample represents a biased (self-selected) population, and our conclusions almost certainly do not apply to asthma patients in general. While this study focuses on asthma patients, the objective of this study was to evaluate the feasibility of using SMS as an interface between the patient and disease, and we believe that our findings may also be valid for similar (self-efficacious) groups of patients with other chronic diseases.

### Diary Usage

We regard a median response rate per patient over 3 months of 0.69 as a positive result. But most importantly, we found that day SMS diary usage did not seem to decline during the study period, which is a common experience both from clinical practice and from previous studies. In our recent study of LinkMedica Asthma, we found that although patients and doctors appreciated the Web-based asthma diary, the use of the Web diary declined rapidly over time, mainly due to poor integration of Internet use into users' everyday life [4].

In this study, the response rate per patient was either below 40% or above 60%, which suggests the existence of two distinct groups of users: high compliers and low compliers. Three of the four low compliers also did not attend the focus group meeting suggesting a relationship between modest overall motivation for participation in the study and low response rate. However, the study was not designed with user segmentation in mind, and this observation should be investigated further in studies with higher sample sizes and a more representative study population.

Contrary to what we expected, the response rate did not seem to be related to the number of messages needing a response each day. Although the participants complained about the number of messages requiring a response, there were only 31 days of incomplete diary data compared to 423 days with complete data.

### SMS Messages as Interface Between Patient and Disease

The results from the focus group interview indicate that the participants did not regard themselves as sick; their chronic asthma was regarded as a condition of life. In relation to this, it is important that any interface between the patient and the disease does not itself make the patient feel sick. However, this does not seem to be the case as expressed by this participant:

Patient 13: * “I was participating in a study [another study] on an Internet data collection system and someone asked me if I felt sicker due to the focus that the study put on my asthma? Certainly not – I feel that I am in control.”*

Interestingly, the participants seemed to make a distinction between technological support and personal support, and they noted that in some cases the support given by a computer system might be superior to in-person support from a health care professional. The attention from another person is, in some situations, felt to be more a burden than help, whereas information technology may provide informal support helping them to gain control without criticism if the user “breaks the rules.” This is in agreement with previous findings by Lange and colleagues who found greater effect from Internet-based psychotherapy of post-traumatic stress than from traditional in-person therapy [12,13].

Most participants in this study truly adopted the SMS diary. It became a part of their disease management, and it gave them a sense of control over the disease, providing more freedom. Looking at the patients' perspective on their asthma disease, it seems as if SMS supports the patients with regard to most of the negative issues of the asthma disease (routines, control). At the same time, it helps them to reach their targets (remembering medication, asthma monitoring). The mobile phone seems to be a good solution to support this process because the mobile phone can easily become an integral part of people's everyday life, not only for traditional phoning but also as a personal management system and information provider. Even though some of the participants had not used SMS prior to this study, the good response rates and the interview showed that this technology was not an obstacle.

### Future Perspectives

In our opinion, the main advantages of SMS diary data collection over traditional Web-based diaries are that SMS is (or easily becomes) an integral part of people's everyday lives, and that SMS includes instant reporting to a central server, which is usually not the case when using, for example, PDAs for data collection and feedback [4,14-18].

The relatively good and, most importantly, stable response rates observed in this study open opportunities for optimizing patient self-care in management of chronic diseases and possibly also for data collection of clinical data during clinical trials.

For the future development and integration of SMS into self-managed asthma care, it is of crucial importance to regard patients as individuals with individual needs. A high degree of system customization is needed. It is also important to recognize the limitations of SMS and mobile phones. The screen and memory size of mobile phones are usually small compared to PDAs and personal computers. Thus, integration with an Internet service that stores and reviews data and makes it possible to customize the mobile phone service is needed as a back-end information hub where the mobile phone serves as the front-end daily communication interface.

We expect that new mobile technologies will appear in the near future, and as these technologies become commonly available, the integration of traditional Web pages with multimedia applications and mobile devices will provide many opportunities for self-management applications in health care.

### Conclusion

The primary objective of this study was to evaluate the feasibility of using SMS for asthma diary data collection from a user perspective. The secondary objective was to investigate response rates of SMS collection of asthma diary data in motivated and self-selected asthma patients.

We conclude that SMS collection of asthma diary data is feasible, and that SMS may be a tool for supporting self-management of asthma (and possibly other chronic diseases) in motivated and self-efficacious patients because mobile phones easily become integrated into people's everyday lives. Thus, the combination of SMS data collection with a traditional Web page for data display and system customization may solve previously addressed problems of poor compliance with Web-based asthma diaries.

## Abbreviations

GPRS: General Packet Radio Service
PDA: Personal Digital Assistant
SMS: Short Message Service
WAP: Wireless Application Protocol
